# Knowledge of Food–Drug Interactions among Medical University Students

**DOI:** 10.3390/nu16152425

**Published:** 2024-07-26

**Authors:** Małgorzata Jelińska, Agnieszka Białek, Małgorzata Czerwonka, Dorota Skrajnowska, Agnieszka Stawarska, Barbara Bobrowska-Korczak

**Affiliations:** 1 Department of Toxicology and Food Science, Faculty of Pharmacy, Medical University of Warsaw, Banacha 1, 02-097 Warsaw, Poland; 2 School of Health and Medical Sciences, University of Economics and Human Sciences in Warsaw, Okopowa 59, 01-043 Warsaw, Poland; 3 The Kielanowski Insitute of Animal Physiology and Nutrition, Polish Academy of Sciences, Instytucka 3, 05-110 Jabłonna, Poland

**Keywords:** food–drug interactions, knowledge, students of pharmacy, medicine, dietetics

## Abstract

Background: Food–drug interactions (FDIs) may alter drug pharmacokinetics and pharmacodynamics, modifying the whole therapy’s effectiveness. Some of them cause the attenuation of drug effects, while others inhibit the medicines’ metabolism resulting, in too high concentrations of the medicine in the body. Thus, some healthcare professionals—doctors, pharmacists or dieticians—should be aware of the possibility of food–drug interactions. This study aimed to assess knowledge of food–drug interactions among students of pharmacy, medicine, stomatology, medical analysis and dietetics and students of the college of further medical education for pharmacy technicians. Methods: Students (n = 820) completed a custom-made questionnaire. The relationships between the continuous variables were analysed on the basis of Pearson’s correlation coefficient. To verify the predictors of objective students’ knowledge about food–drug interactions, a multiple linear regression model with analysis of covariance (ANCOVA) was used. The Kruskal–Wallis test was performed to compare the total scores the respondents received for questions assessing their objective knowledge of FDI. Results: Students’ objective knowledge of FDIs correlated positively with their year of study and their self-evaluation of it. It was also significantly influenced by the field and mode of studies and by the fact that the issue had been discussed during the courses. Conclusions: Most students of the medical university had serious deficiencies in their knowledge of food–drug interactions. This is of particular concern for future doctors and dentists. Among the respondents, pharmacy students stood out, as their FDI knowledge was greater. The issue of food–drug interactions should be more widely taught at medical universities, which was emphasised by the respondents themselves.

## 1. Introduction

In times of common access to medicines, sometimes long-taken and in many cases available without a prescription (over-the-counter drugs, OTC), ergo without a doctor’s supervision, food–drug (FDIs) or drug–nutrient interactions (DNIs) become an extremely important issue. They occur as a result of the concomitant intake of drugs and specific nutrients in food. Drug–nutrient interaction was defined as a pharmacokinetic or pharmacodynamic alteration of a drug or nutrient [1]. Another broader definition was proposed by Boullata [2]. According to that definition, drug–nutrient interaction is a result of “physical, chemical, physiologic or pathophysiologic relationship between a drug and a nutrient, multiple nutrients, food in general or nutritional status” [2]. It may cause changes in the pharmacokinetics and pharmacodynamics of the medicine or in its therapeutic efficacy [3]. Food may affect drugs’ bioavailability, which is an important pharmacokinetic parameter, by influencing gastric acidity and emptying as well as gastrointestinal motility [3]. It can also modify the activity of transport proteins such as *p*-glycoprotein (*p*-gp) in enterocytes of the intestinal villi or metabolising enzymes such as cytochrome *p*-450. These actions can lead to the inhibition or potentiation of drug absorption, distribution, metabolism and excretion from the body, resulting in drug side effects [4]. On the other hand, the reverse direction of food–drug interaction is also possible because drugs, especially when chronically taken, may affect nutrients’ distribution in the body, leading to deficiencies in them [5].

Interactions between drugs and some foods have been reported [3]. The interaction between tyramine-containing foods and monoamine oxidase (MAO) inhibitors is one of the most important and best-known examples. It is called the “cheese reaction”, “cheese effect” or “tyramine reaction” [6]. Tyramine, histamine, putrescine or cadaverine are biogenic amines and are found in ripened cheeses, such as camembert, brie or cheddar, in goat’s or sheep’s milk cheeses, in smoked, salted and marinated fish, in chianti wine and beer. They are also present in some fruits such as avocados, figs and overripe bananas, cured meat and chocolate. Normally, in healthy people, tyramine and other biogenic agents are metabolised by MAO in the intestine and liver. However, the concomitant intake of even one of these products with MAO inhibitor drugs (e.g., isoniazid, furazolidone, procarbazine or antidepressants) leads to increased blood tyramine levels. This causes the stimulation of the adrenergic system with the release of adrenaline from the adrenal glands and noradrenaline from nerve endings. It results in a sudden rise in blood pressure, headaches, retinal pain, visual disturbances or hyperthermia. Symptoms also include restlessness, anxiety, neck stiffness, chills, sweating, vomiting and collapse. In extreme cases, heart failure and infarction, haemorrhages, stroke or comas can occur. Ingestion of about 6 mg of tyramine with a MAO inhibitor can lead to death [6,7].

Grapefruit juice is probably a best-known reason for food–drug interactions [8]. It contains 6,7-dihydroxybergamottin, belonging to furanocoumarins and flavonoid naringin. Both of these compounds as well as the naringin aglycone naringenin inhibit cytochrome P450 CYP 3A4 activity in the small intestine and in the liver and to some extent other isoforms of this enzyme [9]. It results in the increased absorption and reduced metabolism of drugs leading to increased concentrations of them. Interactions with grapefruit juice are particularly dangerous because of the type of drugs whose metabolism is inhibited. These include calcium channel blockers, hypolipemic drugs, antihistamines and antivirals used to treat AIDS [10,11,12,13].

High intake of vitamin K, either in the form of medicines or supplements, or vegetables rich in it, inhibits the effectiveness of anticoagulant drugs such as warfarin [14,15]. Dietary fibre, found in oat flakes, can bind various drugs, reducing their absorption and consequently their effectiveness, which can often be the cause of dangerous health effects. Fibre’s interaction with digoxin may cause circulatory failure or arrhythmia while its interaction with antidepressants can be life-threatening for people with depression [16].

As can be seen, food–drug interactions can significantly affect the course and efficacy of therapy. However, this issue is still not sufficiently understood because studies in this area are difficult to conduct and, therefore, many data are simply missing. It appears that pharmacists and physicians can play a special role here by providing the patient with information on how to take a drug. Moreover, pharmacists are often the first healthcare professionals a patient goes to for help and advice. Their knowledge of food–drug interactions seems to be essential for therapy efficacy and avoiding drug side effects.

The aim of this study was to assess the knowledge of food–drug interactions among the students of pharmacy, medicine, stomatology, medical analysis and dietetics as well as the students of the college of further medical education for pharmacy technicians. The study also aimed to analyse which aspects of students’ educational path influence their knowledge about FDIs and what the relative contribution of these respective predictors to this knowledge is. We were also interested in what role the students think FDIs might play in their future work and whether, at the current stage of their studies, they are aware of the risks that may result from food–drug interactions. None of the studies we know of have analysed such a diverse group of respondents taking into account a broad cross-section of healthcare disciplines. In this regard, our paper significantly fills a gap in the literature.

## 2. Materials and Methods

### 2.1. Participants

The study involved students of pharmacy, medicine, stomatology, medical analysis and dietetics, as well as of the college of further medical education for pharmacy technicians. A total of 820 respondents participated in the study.

### 2.2. Procedure

The study was conducted during the second term of the academic year in paper–pencil form. A custom-made questionnaire was given to the students during their courses. Given the limited length of the questionnaire, its completion took from 10 to 15 min. The respondents were informed about the purpose of the survey and they participated anonymously and voluntarily.

### 2.3. Measures

A custom-made survey, composed of 21 items, was designed to verify students’ subjective and objective knowledge of food–drug interactions as well as the application of them in their own life.

Objective knowledge of the interactions was assessed using four items. In three of them, the participants were asked to indicate one correct answer concerning their acquired knowledge about existing interactions between (1) vitamins and anticoagulant drugs, (2) drugs from a group of monoamine oxidase inhibitors (MAOIs) and different types of food, and (3) grapefruit juice consumption and the time interval while taking drugs. One question asked (4) to give an example and brief characteristics of one food–drug interaction familiar to the students. The participants received one point for each correct answer. The calculated sum of points for all questions gave insight into the students’ objective knowledge about food–drug interactions.

Subjective knowledge was assessed by Likert type items (ranging from 1—“never” to 5—“always”). The exploratory factor analysis (EFA) allowed researchers to distinguish two factors, which constitute two separate subscales:

- Self-application of knowledge about food–drug interactions was assessed by three items measuring to what extent students themselves applied their knowledge about food–drug interactions while personally taking drugs;
- Food–drug interaction awareness was assessed by two items measuring to what extent students are aware of existing interactions between drugs and foods. Due to the fact that both scales are short, and to make them equinumerous, an item was added to this scale.

The single-item indicators were used to measure the self-evaluation of knowledge about food–drug interactions, showing to what extent students found their knowledge to be basic, intermediate, advanced or proficient, the evaluation of FDI knowledge sufficiency, indicating whether students found their current knowledge to be satisfactory and sufficient or the need to improve it, and FDI knowledge usefulness in future professional settings, reporting whether students thought that kind of knowledge would be necessary and useful in their future work. A single-item indicator was also used to assess whether there is a need among students to learn more about food–drug interactions in the form of a separate subject included in their curricula or as a topic of one particular class. Apart from these items, we also collected basic sociodemographic information such as students’ age, gender, faculty, year and mode of studies.

### 2.4. Data Analysis

Statistical analysis was conducted with STATISTICA 13.3 (TIBCO Software Inc., Palo Alto, CA, USA). The Kruskal–Wallis test, which is a non-parametric version of an analysis of variance, followed by Dunn’s test was used to compare the total scores the respondents received for questions assessing their objective knowledge of FDIs. The relationships between the continuous variables were analysed on the basis of Pearson’s correlation coefficient. To verify the predictors of students’ objective knowledge about food–drug interactions, we used the STATISTICA’s General Regression Models (GRM) module, which applies the methods of the general linear model allowing us to build models combining categorical and continuous predictor variables (an analysis of covariance design). The significance level was set at 0.05 for the Pearson’s correlations and Kruskal–Wallis’ test and 0.001 for the multiple linear regression model. Effect sizes are reported with *η*p^2^ for AN(C)OVA-based analysis.

To test the linearity assumption, a visual inspection of scatterplots was conducted which indicated that the variables and the residuals of the regression (i.e., the errors between observed and predicted values) were normally distributed. Multicollinearity was verified by the variance inflation factor (VIF) and tolerance. The VIF value for the variables included in the model in the case of one variable slightly exceeding three, whereas the tolerance value ranged from 0.31 to 0.98. These indicators confirm a lack of collinearity. It was also proved with the matrix of Pearson’s bivariate correlations among all predictors. The correlation coefficients were lower than 0.80 (the highest correlation obtained in a single case was r = 0.69) which proved that there was no multicollinearity in the data. The homoscedasticity assumption was verified visually using a scatterplot of residuals versus predicted values and was also met.

## 3. Results

In this study investigating students’ knowledge about food–drug interactions (FDIs), a total of 820 students took part. The overwhelming majority of them, i.e., approximately 93% of them, studied one of five majors: pharmacy, medicine, stomatology, medical analysis and dietetics, whereas 7% pursued their studies in the college of further medical education for pharmacy technicians (CPhT). Among all these fields of study, pharmacy (33.9%) and medicine (30.6%) were the most represented (Table 1). As many as 91.3% of the participants declared themselves to be full-time students. The respondents represented all stages of study, from the first to the fifth year. In general, 73% of participants were in their second or third year of study. Among the students, women accounted for more than 75% and 68% were aged from 21 to 25 (Table 1).

**Figure 1:**
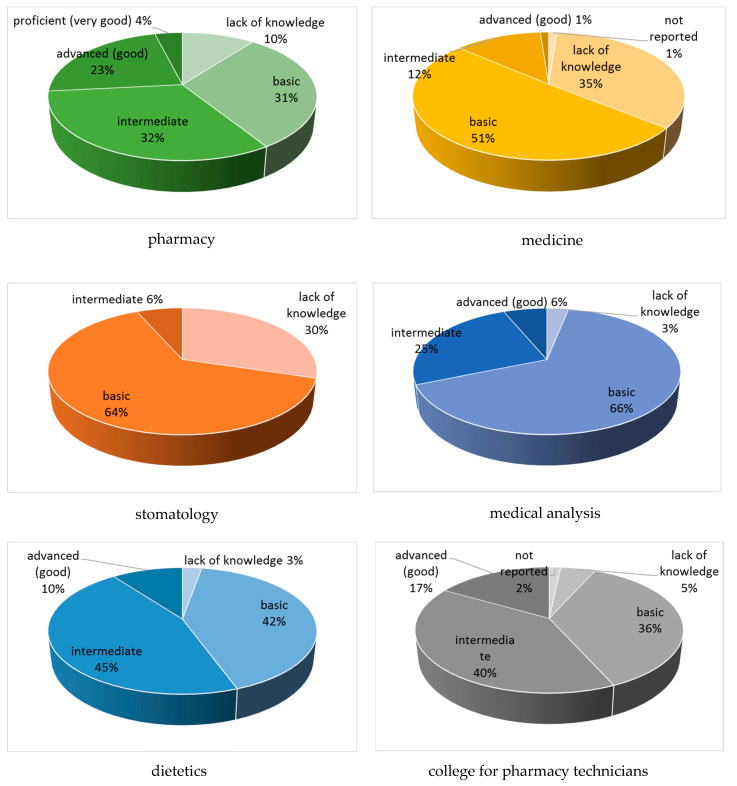
Self-evaluation of FDI knowledge depending on the field of study.

More than 25% of students declared that a patient information leaflet was their first source of knowledge about food–drug interactions. Approximately 24% of the participants heard about this issue for the first time during their studies, whereas 14% indicated they read about it in popular science reviews (Table 1). More than half the students (54%) reported that the matter of food–drug interactions should be taught in the form of a separate subject instead of as one of the topics mentioned briefly during one course. Approximately 44% of them found their knowledge about food–drug interactions to be basic, contrary to the 12% reported to have good or very good knowledge of this issue. Furthermore, only a few pharmacy students assessed their FDI knowledge as proficient (very good), which constitutes 4% of students in this field (Figure 1). They were third- and fourth-year students. Among medical students, only just over 1% considered their knowledge of interactions to be good, while 35% of them reported no knowledge of the subject. Similarly, 30% of stomatology students admitted to having no knowledge of the issue at all (Figure 1). Finally, the overwhelming majority, i.e., 90%, estimated that the knowledge about food–drug interactions may be useful in their future professional activities. Table 1 presents all the sociodemographic characteristics of the students.

The survey was performed to estimate students’ objective and subjective knowledge of drugs interactions with foods. Objective knowledge was measured by answering four questions for which each respondent could obtain a total of 4 points—a point for each question.

The predominant number of students surveyed (608; 74%) answered the question about interactions of anticoagulant drugs with food correctly (Table 2). Within this group, pharmacy students accounted for 38%, followed by students of medicine (31%) and dietetics. Among a particular field of study, correct answers to this question constituted over 80% in the case of pharmacy (84%) and dietetics (82%) students, 75% in the case of medicine students. A summary of all correct answers to this question is shown in Table 2.

Contrary to the previous question, students’ knowledge of interactions between monoamine oxidase inhibitors and foods seemed to be much worse. Less than 34% of students correctly answered the question of what foods to avoid when taking MAOIs (Table 3). Similarly to the previous question, the pharmacy students were more aware of this type of interaction compared to other participants—about 65% of them indicated the correct answer (Table 3).

They were followed by the medicine students—but only less than 18% of them marked the correct answer. Unfortunately, as many as 43% of the respondents did not know the correct answer to this question and the medicine students accounted for 42% (Table 4). Among medical students, 60% did not know the correct answer to the question about MAOI interactions with foods. The answer ‘I do not know’ was also chosen by as many as 70% of stomatology students (Table 4).

Regarding the question about the effect of grapefruit juice on drug biotransformation, the results indicated that only 38% of the respondents of all study fields knew how long the time interval between consuming grapefruit juice and taking drugs should be (Table 5). Similar to the previous questions, the pharmacy students gave most of the correct answers (49%), followed by the medical students (20%). In the particular fields of study, the future pharmacists also indicated most of correct answers (nearly 56%) (Table 5).

Total scores in students’ objective knowledge of food and drug interactions are shown in Table 6. The pharmacy and dietetics students received the highest scores. For pharmacy students, the result was significantly higher when compared to the scores obtained by students of each of the other fields of study. It accounted for 69% of the maximum score. The score obtained by the dietetics students was significantly increased in comparison to the results achieved by the students of medicine, stomatology and the college for pharmacy technicians. It constituted 58% of the point value (Table 6). The scores received by the students of medicine, stomatology, medical analysis and the college for pharmacy technicians were less than 50% of the maximum value of points.

The results of Pearson’s correlation analyses (Table 7) show the relationships between objective knowledge about food–drug interactions and the other variables. Students who presented higher objective knowledge about drug and food interactions evaluated their knowledge thereof as higher (r = 0.49). The level of their objective knowledge on this topic was also strongly related to their year of study (r = 0.55). Interestingly, the year of study strongly positively correlated with students’ self-evaluation of their knowledge about food–drug interactions (r = 0.51) which, in turn, was related to the first time the student heard about food–drug interactions during their studies (r = −0.42). This means that the earlier the students heard about the interactions between drugs and foods, the better their objective results were.

To find out which factors and to what extent they determine students’ objective knowledge about food–drug interactions, an eight-step multiple linear regression model based on forward selection using an ANCOVA design was built (Table 8).

The results, provided in Table 8, indicate that students’ objective knowledge about food–drug interactions depends most on the year of study (β = 0.36, t = 7.57, *p* < 0.001). It is worth of noticing that the influence of this predictor was more than twice as strong as that of the other variables. Partial eta2 indicates that the year of study predicted 7% of the variance in students’ objective knowledge about food–drug interactions, which constitutes a medium size effect. The other important predictors of what students objectively know about interactions between drugs and food was their FDI knowledge self-evaluation and, in particular, whether they found it to be sufficient or insufficient (β = 0.18, t = 4.92, *p* < 0.001). It was also determined by the mode of study with the significant effect given by full-time study (β = 0.12, t = 3.70, *p* < 0.001). The next important predictor of the objective FDI knowledge turned out to be the faculty (respectively pharmacy β = 0.23, t = 5.01, *p* < 0.001; medicine β = 0.11, t = 2.87, *p* < 0.001 and the college of further medical education β = −0.11, t = −2.21, *p* < 0.05; stomatology and medical analysis had no significant effects). Students’ objective knowledge about food–drug interactions depended also on whether this topic had been already discussed during their courses or not (β = 0.13, t = 3.42, *p* < 0.001). Finally, it was also predicted by the extent to which students themselves applied their knowledge about food–drug interactions while personally taking drugs (β = 0.07, t = 2.24, *p* < 0.05), which was immediately followed by students’ self-evaluation indicating whether their current knowledge about food–drug interactions is sufficient or not (β = 0.06, t = 2.13, *p* < 0.05). Interestingly, the aspect of subjective knowledge such as food–drug interaction awareness, as well as the first source of the knowledge about FDIs (i.e., studies or patient drug leaflets), students’ age and gender were the factors which did not contribute to the regression model.

The entire regression model is significant (F11,725 = 46.60, *p* < 0.001) and predicts 41% of variance in students’ objective knowledge about food–drug interaction (Table 9). Moreover, partial eta2 shows that its size effect is large (*η*_p_^2^ = 0.41) which indicates that the factors determining students’ objective knowledge analysed in our study are of great importance at more general level.

**Figure 2:**
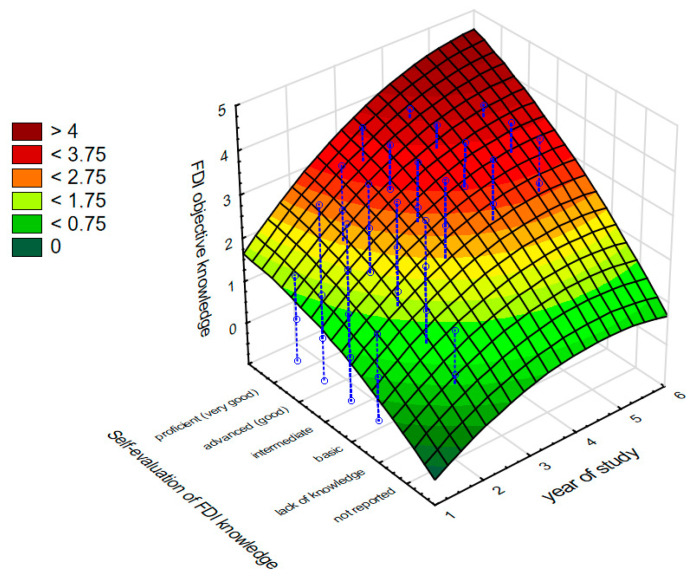
The effect of the years of study and students’ self-evaluation of their knowledge about food–drug interactions on their objective knowledge about food–drug interaction.

Figure 2 shows the effect of the most important predictors such as the year of study and the students’ self-evaluation of their knowledge about food–drug interactions on what they objectively knew about this issue.

## 4. Discussion

Food–drug interactions may result in alterations in drug pharmacokinetics and pharmacodynamics and thus modify the effectiveness of the entire therapy [17,18]. Some of them cause the attenuation of drug effects, while others are associated with the inhibition of the medicines’ metabolism and consequent adverse effects resulting from a too-high concentration of the drug in the body. Thus, as can be seen, some healthcare professionals—physicians, pharmacists or dieticians—should be aware of the possibility of food–drug interactions.

The aim of our study was to assess the knowledge of food–drug interactions among students of pharmacy, medicine, stomatology, medical analysis and dietetics, as well as students of the college of further medical education for pharmacy technicians. We wanted to find out which aspects of the students’ educational path influence their knowledge of food–drug interactions and what the relative contribution of these predictors to the students’ knowledge is. We were also interested to know what role the students think FDIs might play in their future work and whether, at the current stage of their studies, they are aware of the risks that may result from food–drug interactions.

The survey indicated the students’ knowledge of food and drug interactions was deficient. It was found that as many as 18% of those surveyed admitted to having no knowledge of the subject at all. A total lack of FDI knowledge was declared by 35% and 30% of medicine and stomatology students, respectively, as well as by 10% of pharmacy students, 5% of students of the college for pharmacy technicians and 3% of both dietetics and medical analysis students. While this may be less of a problem in the case of medical analysts as they have no direct contact with patients, the lack of knowledge about food–drug interactions raises serious concerns if it involves physicians, pharmacists, pharmacy technicians as well as dentists. Our results may be explained by the respondents’ years of study. More than 50% of them were second-year students, while the statistical analysis of the findings indicated a very strong relationship between students’ knowledge and their year of study. The maximum score for questions testing the students’ objective knowledge of FDIs was equivalent to 69.5%. This result was achieved by the pharmacy students. It was followed by 58%, the value obtained by the future dieticians. Unfortunately, students of other majors scored less than 50%, confirming their knowledge deficiencies. Our results are in agreement with several other works that revealed gaps in knowledge about FDIs among physicians, pharmacists and nurses [19,20,21,22]. In these studies, the mean percentage scores were about 61%. In this context, research by Jyoti stands out [19]. They included Indian physicians with varying degrees of education. They found that those with a professor title scored nearly 84% in the test, indicating their very good knowledge of the issue of food–drug interactions. In turn, Radwan et al. noted that pharmacists’ knowledge of FDIs is closely related to their years of work and experience [20]. Similarly, Enwerem et al. observed significant differences in food and drug interaction knowledge among nurses, depending on their working experience [22].

Generally, our research indicated the pharmacy students had presented a more thorough knowledge of food and drug interactions. As much as 4% of them rated their knowledge as very good, while 23% as rated theirs as good, a score that was not achieved by students from other fields of study. They were more aware of interactions compared to participants representing the other fields of study. About 65% of pharmacy students indicated the correct answer to the question of what foods to avoid when taking MAOIs. The difference in knowledge between the future pharmacists and the other respondents was alarming. Only about 20% of students of medicine, dietetics and the college for pharmacy technicians answered this question correctly. In this respect, the results of our survey are also in complete agreement with other studies in which practising pharmacists and physicians have participated [19,20]. About 72% of pharmacists involved in Palestinian research were aware of the interaction of MAOIs, used in the treatment of depression, with foods containing tyramine, such as cheese. Small amounts of this normally harmless amin taken with monoamine oxidase inhibitors may lead to a life-threatening hypertensive crisis in the patients [6]. In contrast to the pharmacists but in total agreement with our findings, medicine interns participating in the Indian study showed a lack of knowledge of the interaction of tyramine-containing foods with MAOIs [19]. Postgraduates appeared to be slightly better, whereas professors of medicine turned out to be aware of tyramine—MAOI interactions.

Statistical analysis indicated that the students’ objective knowledge of food–drug interactions also correlated positively with their self-evaluation and the field and mode of study. It was influenced by the fact that the issue had been discussed during their courses. However, similar to other studies, the students’ age and gender, as well as their first source of knowledge about FDIs (i.e., studies or patient drug leaflets) did not affect their knowledge of food and drug interactions [20].

Approximately 44% of the students participating in the survey found their knowledge of food–drug interactions to be basic, while only 12% reported to have good or very good knowledge of this issue. Finally, the overwhelming majority (i.e., 90%) of the respondents of various fields of study thought that the knowledge of food–drug interactions may be useful in their future professional activities. More than half of them (54%) reported that the issue of food–drug interactions should be taught in the form of a separate subject instead of as one of the topics mentioned briefly during one course. Such good results of pharmacy students in the questionnaire may be explained by the presence of the subject “Bromatology”, i.e., food and nutrition science, among the compulsory subjects in the field of pharmacy. The bromatology curriculum emphasises the issue of drug–food interactions. More complex education would develop students’ knowledge and diminish the occurrence of drug interactions.

The strength of the study is that it gives lecturers an insight into what the various healthcare students actually know about FDIs and to what extent they are aware of their knowledge. The aspect of subjective knowledge, i.e., being aware of what one knows, is very important because it allows students and later graduates to consciously use this knowledge in their professional practises when interacting with patients. Subjective knowledge makes it easier to extract and apply the objective knowledge acquired in class. For lecturers, the results of our study are very important, allowing them to perceive the discrepancy between objective and subjective knowledge and draw attention to the necessity of developing subjective knowledge among students, and therefore are a starting point for changing or adapting the form of teaching, so that students, in addition to their objective knowledge, are also aware of having knowledge at an appropriate level.

The limitation of the study is that all data were collected solely with a questionnaire, and were therefore generalised and constrained only to the questions strictly specified in the survey. This did not allow individual differences to be taken into account, which would have emerged in a qualitative study using a structured or semi-structured interview. Additional limitations also result from solely using solely the paper-and-pencil technique, which did not give the opportunity to reach an even wider range of students from other universities as well. Finally, it would be worthwhile to have more control over the field of study variable and to design the study in such a way that the groups are equal, as this will give more insight into the differences among the students of different fields of study and the level of the curriculum as well.

## 5. Conclusions

Most students examined had serious deficiencies in their knowledge of food–drug interactions. This is of particular concern for future doctors and dentists. Among the respondents, pharmacy students stood out, as their FDI knowledge was greater. The issue of food–drug interactions should be more widely taught at medical universities, which was emphasised by the respondents themselves. It should be complemented in particular in the curriculum of fields of study such as medicine, dentistry and dietetics and further developed in the field of pharmacy. Future research should include an online version of the survey, which could be accessible to a wider range of medical university students across the country or even worldwide. In order for the survey itself to be of direct benefit to the participants and therefore to build their subjective knowledge of food–drug interactions, it would be worthwhile to enrich the study with a qualitative component, which would be conducted in focus groups using discussions or a problem-based learning method.

## Figures and Tables

**Table 1:** Sociodemographic characteristics of the participants (*N* = 820).

	Frequency (n)	Percent (%)
**Field of study**		
pharmacy	278	33.9
medicine	251	30.6
dietetics	108	13.2
stomatology	91	11.1
college for pharmacy technicians (CPhT)	60	7.3
medical analysis	32	3.9
**Mode of studies**		
full-time	749	91.5
part-time	70	8.4
not reported	1	0.1
**Gender**		
female	616	75.1
male	199	24.3
not reported	5	0.06
**Age groups (years)**		
<20 years		25.6
21–35		68.0
>25 years		4.3
not reported		2.1
**Year of studies**		
1	64	7.8
2	436	53.2
3	171	20.8
4	113	13.8
5	36	4.4
**First source of knowledge about FDI**		
patient information leaflet	208	25.4
study courses	195	23.8
popular science reviews	116	14.2
other	301	36.6
**Need for teach FDI as separate subject**		
yes	445	54.3
no	161	19.6
without preferences	211	25.7
not reported	3	0.4
**Self-evaluation of FDI knowledge**		
lack of knowledge	150	18.3
basic	358	43.7
intermediate	208	25.4
advanced (good)	89	10.8
proficient (very good)	11	1.3
not reported	4	0.5
**FDI knowledge usefulness in future work**		
yes	738	90
no	17	7.9
do not know	65	2.1

**Table 2:** Students’ knowledge of interactions between foods and anticoagulant drugs expressed as the number and percentage of correct answers.

Field of Study	Correct Answers		
	Frequency (*n*)	Percent of Students in a Field of Study (%)	Percent of All Correct Answers (%)
Pharmacy	235	84.5	38.6
Medicine	189	75.3	31.1
Stomatology	50	54.9	8.2
Medical analysis	19	59.0	3.1
Dietetics	89	82.4	14.6
College for pharmacy technicians (CPhT)	26	43.3	4.3
All students	608	–	74.1

**Table 3:** Students’ knowledge of interactions between drugs from a group of monoamine oxidase inhibitors (MAOIs) and different types of foods, expressed as the number and percentage of correct answers.

Field of Study	Correct Answers		
	Frequency (n)	Percent of Answers in a Field of Study (%)	Percent of All Correct Answers (%)
Pharmacy	181	65.1	65.6
Medicine	49	19.5	17.8
Stomatology	9	9.9	3.3
Medical analysis	2	6.2	0.7
Dietetics	22	20.4	8.0
College for pharmacy technicians (CPhT)	13	21.7	4.7
All students	276	–	33.7

**Table 4:** The number and percent (%) of students who did not know the correct answer to the question concerning MAO inhibitor interactions with foods and who chose I do not know in the survey.

Field of Study	“I do not know” Answer		
	Frequency (n)	Percent of Answers in a Field of Study (%)	Percent of Answers (%)
Pharmacy	63	22.7	18.0
Medicine	148	59.0	42.4
Stomatology	64	70.3	18.3
Medical analysis	15	46.8	4.3
Dietetics	41	38.0	11.7
College for pharmacy technicians (CPhT)	18	30.0	5.2
All students	349	–	42.6

**Table 5:** Students’ knowledge of the time interval between grapefruit juice and drug intake, expressed as the number and percentage of correct answers.

Field of Study	Correct Answers		
	Frequency (n)	Percent of Answers in a Field of Study (%)	Percent of All Correct Answers (%)
Pharmacy	155	55.8	49.0
Medicine	64	25.5	20.2
Stomatology	12	13.2	3.8
Medical analysis	16	50.0	5.1
Dietetics	48	44.4	15.2
College for pharmacy technicians (CPhT)	21	21.7	6.6
All students	316	–	38.5

**Table 6:** Total scores in students’ objective knowledge of food–drug interactions.

Field of Study	Mean Score	Percent (%)
Pharmacy	2.78 ± 0.07 ^abc^	69.5
Medicine	1.64 ± 0.06 ^a^	41.0
Stomatology	1.10 ± 0.09 ^a^	27.5
Medical analysis	1.69 ± 0.18 ^b^	42.2
Dietetics	2.32 ± 0.10 ^ac^	58.0
College for pharmacy technicians (CPhT)	1.28 ± 0.15 ^c^	32.0

Data were expressed as mean ± standard error of mean (SEM). Values in a column sharing the same index are significantly different (*p* < 0.05).

**Table 7:** Pearson’s *r* correlation coefficients between objective knowledge about food–drug interactions and the other variables (*N* = 820).

	1	2	3	4	5	6	7
1. Objective knowledge about FDIs							
2. FDI awareness	0.05						
3. FDI self-application	0.19 *	−0.03					
4. FDI knowledge sufficiency	0.03	0.03	0.01				
5. FDI knowledge self-evaluation	0.49 *	0.03	0.23 *	−0.04			
6. FDIs taught during studies (years of study)	−0.27 *	−0.05	−0.18 *	−0.04	−0.42 *		
7. Years of study	0.55 *	−0.01	0.13 *	−0.01	0.51 *	−0.31 *	
8. Age	0.15 *	0.08 *	0.11 *	−0.01	0.23 *	−0.28 *	0.21 *

* *p* < 0.05. Numbers in the headline correspond to variables tested (1—objective knowledge about FDI, 2—FDI awareness, 3—FDI self-application, 4—FDI knowledge sufficiency, 5—FDI knowledge self-evaluation, 6—FDI taught during studies (years of study), 7—years of study, 8—age).

**Table 8:** Eight-step multiple linear regression model with ANCOVA (forward selection) for variables predicting students’ objective knowledge about food–drug interactions.

Step	Independent Variables	*b*	SE	*β*	*t*	*R* ^2^	*F*	*η*_p_^2^	95%CI	
1	Years of study	0.45	0.06	0.36 **	7.57	0.64	57.31	0.07	0.05	0.11
2	FDI knowledge self-evaluation	0.25	0.05	0.18 **	4.92	0.41	24.17	0.03	0.01	0.06
3	Mode of study (full-time)	0.26	0.07	0.12 **	3.70	0.18	13.67	0.02	0.01	0.04
4	faculty						8.22	0.05	0.03	0.08
	Pharmacy	0.41	0.08	0.23 **	5.01	0.60				
	Medicine	0.22	0.08	0.11 **	2.87	0.49				
	stomatology	−0.08	0.12	−0.03	−0.69	0.60				
	CFME	−0.31	0.14	−0.11 *	−2.21	0.69				
	Medical analysis	−0.08	0.15	−0.03	−0.56	0.69				
5	FDIs discussed during studies (yes)	0.17	0.05	0.13 **	3.42	0.44	11.69	0.02	0.00	0.03
6	FDI self-application	0.10	0.05	0.07 *	2.24	0.05	5.00	0.01	0.00	0.02
7	FDI knowledge sufficiency	0.18	0.08	0.06 *	2.13	0.02	4.55	0.01	0.00	0.02
−	Gender	−	−	−	−	−	−	−	−	−
−	FDI awareness	−	−	−	−	−	−	−	−	−
	Studies as the first source of FDI knowledge	−	−	−	−	−	−	−	−	−
−	Patient drug leaflet as the first source of FDI knowledge	−	−	−	−	−	−	−	−	−
−	Age	−	−	−	−	−	−	−	−	−

* *p* < 0.05, ** *p* < 0.001.

**Table 9:** The regression results of the effects of measured variables on the students’ objective knowledge about food–drug interactions.

Dependent Variable	*R*	*R* ^2^	Adj. *R*^2^	*df* 1	*df* 2	*F*	*η*_p_^2^	95%CI	
Objective knowledge about FDIs	0.64	0.41	0.41	11	725	46.60	0.41	0.36	0.45

## Data Availability

Data supporting reported results can be received after sending a written request to the corresponding author.
